# Extraction and Identification of Polysaccharide from *Lentinus edodes* and Its Effect on Immunosuppression and Intestinal Barrier Injury Induced by Cyclophosphamide

**DOI:** 10.3390/ijms252212432

**Published:** 2024-11-19

**Authors:** Xiaodi Jin, Zhiyong Wu, Hao Chen, Weiqi Liu, Fuhua Gu, Jichang Li

**Affiliations:** 1 College of Veterinary Medicine, Northeast Agricultural University, Harbin 150030, China; jxd19950925@163.com (X.J.); wuzhiyong@neau.edu.cn (Z.W.); chenhao980915@163.com (H.C.); 17621867263@163.com (W.L.); fhua666@126.com (F.G.); 2 Heilongjiang Academy of Chinese Medicine Sciences, Harbin 150030, China

**Keywords:** lentinan, immunosuppression, intestinal barrier, gut microbiota

## Abstract

*Lentinus edodes* serves as a significant source of both medicine and food, with its key component, lentinan (LNT), recognized as an effective immunomodulator. However, the mechanisms by which it regulates immune and intestinal functions under conditions of immunosuppression remain unclear. This study aims to investigate the components of lentinan and examine its potential effects on countering cyclophosphamide (CP)-induced immunosuppression, intestinal barrier damage, and dysregulation of gut microbiota. In this study, the effects of LNT were evaluated by serological indicators, histopathological changes in ileum, tight-junction-related protein expression, cytokine expression levels, and gut microbiota 16S rRNA gene sequencing. We found that LNT was effective in mitigating the abnormalities in body weight, immune organ index, and serum levels of IL-6, IL-2, IFN-γ, and IgG in mice induced by CP (*p* < 0.05). Furthermore, LNT demonstrated the ability to alleviate intestinal barrier damage induced by CP by increasing the mRNA levels of TNF-α, IL-1β, IFN-γ, Occludin, and ZO-1 (*p* < 0.05). Additionally, 16S rRNA gene sequencing revealed that LNT also normalized the disrupted abundance of *Firmicutes*, *Proteobacteria*, and *Bacteroidets* caused by CP. This restoration brought the gut microbiota back to normal levels and increased the abundance of certain tumor-inhibiting bacteria, such as *Alistipes*. Overall, lentinan demonstrated the ability to reverse the immunosuppressive effects induced by cyclophosphamide and modulate gut microbiota to restore a healthy microbial balance.

## 1. Introduction

In recent times, attention has increasingly turned to the advancement of active compounds within traditional Chinese medicine, especially concerning research on anti-inflammatory properties, antioxidant effects, and immune system functionalities. The immune system is essential for the body, helping to fend off pathogen attacks and maintain internal balance. Once the immune system is compromised, the body becomes more sensitive to pathogens [1]. Cyclophosphamide (CP), a chemotherapy agent, can influence apoptosis mediated by the Fas/Fas L death receptor signal transduction pathway [2] and can also affect cytokine secretion through the NF-κB pathway [3,4]. Furthermore, recent studies have demonstrated that CP can induce an imbalance in gut microbiota intestinal flora [5]. Therefore, it is often used to establish immunosuppressive models [6,7]. At present, the immunomodulators used in clinical practice have certain side effects, such as Levamisole [8]. Therefore, the development of safer immunomodulators is of great significance for the prevention and treatment of immunosuppressive diseases. Contemporary studies in medicine have established that traditional Chinese medicine can exert an influence on the immune system by affecting both innate and acquired immunity [9,10,11].

Over the past decades, immunostimulation has been one of the most exploited biological functions of polysaccharides [12]. *Lentinus edodes*, which originated in China, is the second-largest mushroom produced in the world and is also a precious medical mushroom with a long reputation in China. Lentinan (LNT), the principal active component extracted from *Lentinus edodes*, exists as sugar chains interconnected by glycosidic bonds. It is a high-molecular-weight polymer composed of various monosaccharides. Previous research has been demonstrated to have a strong effect on immunomodulatory [13,14,15], antitumor [16], and antiviral effects [17]. Research indicates that LNT may serve as a natural protective agent against damage in bovine mammary epithelial cells, which are mediated by the modulation of the Nrf2 pathway, owing to its anti-inflammation [18]. Moreover, LNT could improve antibiotic-induced gut microbiota imbalance in mice [19]. Gut microbiota has been extensively studied, as it not only reflects the normal physiological functions of the body but also influences overall health. An imbalance in gut microbiota can lead to chronic immune diseases. Research has demonstrated that such imbalances may also impact neurological conditions, including Alzheimer’s disease, via the gut–brain axis [20]. Consequently, flora plays a crucial part in maintaining healthy immune regulation and intestinal barrier function [21]. Further investigation is necessary to determine if lentinan can reduce the side effects associated with cyclophosphamide, enhance immune function, and modulate gut microbiota.

Therefore, CP was utilized in this study to create an immunosuppressive mouse model for examining the modulation of immune function and the therapeutic impact of LNT on intestinal function and gut microbiota in mice. The aim of this study is to extract and identify the key components of lentinan, investigate its regulatory effects on immunosuppression induced by CP, analyze its impact on intestinal flora, and explore the drug mechanism of lentinan. The findings aim to offer insights into the potential combined use of immunomodulators and microbial agents in future applications.

## 2. Results

### 2.1. Characterization of LNT

**Figure 1:**
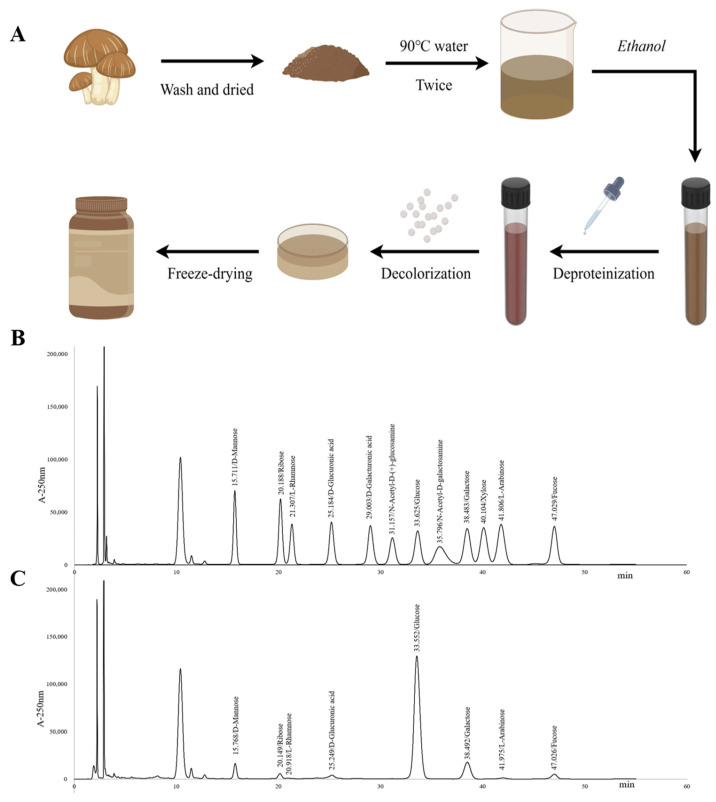
Extraction and HPLC identification of lentinan: (**A**) Extraction process of lentinan. The figure was designed by Figdraw 2.0. (**B**) A chromatogram of standard mixed solution. (**C**) A chromatogram of sample solution.

LNT appeared as a hygroscopic powder with a brownish-yellow hue, with a total carbohydrate content of 73.43% (*w*/*w*) determined using the phenol–sulfuric acid method. Additionally, the composition included 17.17% protein content (*w*/*w*) as measured by a protein assay kit and 3.03% galacturonic acid content. The result of monosaccharide components in Table 1 shows that it was mainly composed of glucose (76.10%), galactose (11.67%), and D-mannose (4.73%). It also contained small amounts of fucose (2.96%), ribose (1.82%), and D-glucuronic acid (6.23%) (Figure 1C). The following supporting information can be found in Supplementary Materials File S1.

**Figure 2:**
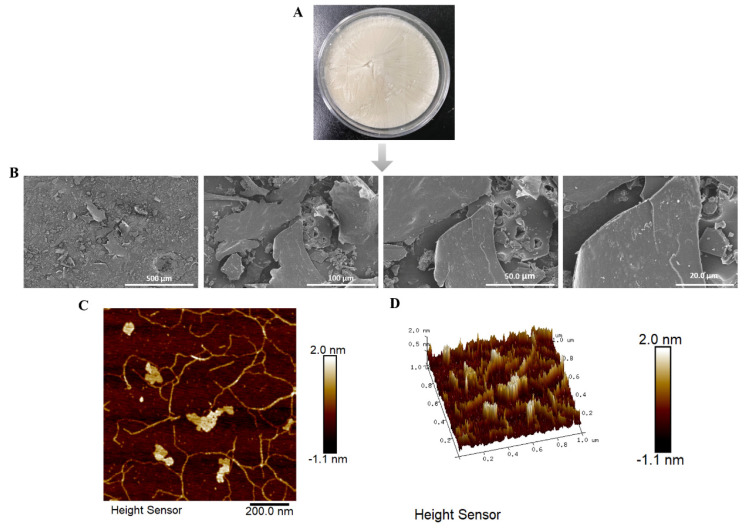
Identification of surface characteristics of lentinan: (**A**) Freeze-dried lentinan. (**B**) The SEM images of LNT were obtained in the order of 500–20 μm. (**C**,**D**) Atomic force microscopy (AFM) 2D, phase and 3D images, respectively.

The results from SEM illustrate the surface morphology of LNT at four distinct magnifications: 500 μm, 100 μm, 50.0 μm, and 20.0 μm (Figure 2B). SEM analysis indicated that LNT exhibited an irregular large layer structure, characterized by a minimal presence of fibrotic structures interwoven with a lamellar arrangement. Atomic force microscopy (AFM) is an effective method for observing the surface morphology of polysaccharides [22]. Atomic force microscopy results in Figure 2C revealed that, in the 2D image, LNT displayed a sheet-like and fibrous structure, with heights ranging from −1.1 nm to 2.0 nm, and the value of Image Ra was 0.273 nm. The 3D image shown in Figure 2D reproduces the same structure as that in SEM.

### 2.2. LNT Regulates Body Weight and Index of Immune Organ

**Figure 3:**
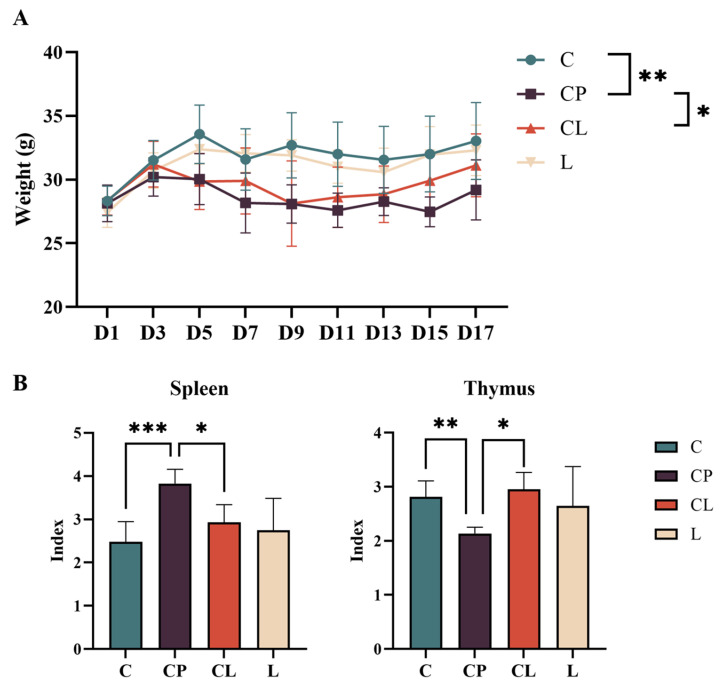
Physical indexes of experimental animals: Effects of CP and LNT on body weight and organ index after administration. (**A**) Body weight curves of four groups. (**B**) The organ indices of mice. Data are presented as the mean ± SD and analyzed by one-way ANOVA with Tukey’s multiple comparisons. * *p* < 0.05, ** *p* < 0.01, *** *p* < 0.001.

To assess the impact of LNT on the immunosuppressive effects caused by CP, an experiment utilizing mice was conducted, illustrated in Figure 3. CP has been shown to have an effect on body weight in mice after intraperitoneal injection [23,24]. Figure 3A, following five consecutive days of CP administration, there was a notable decline in the body weight of the mice subjected to CP. By the fifth day, the body weight in the CP group was significantly lower than that in the C group (*p* < 0.05). After 9 days, an observed gradual increase in body weight was noted in the CL group. On day 17, the body weight of C-group mice was significantly higher than that of the CP group (*p* < 0.05). No notable difference in body weight was found between the mice treated with LNT and those in the control group.

Immune organs can reflect the immune function of the body [25]. After the administration of CP, both the thymus and spleen displayed significant changes. In Figure 3B, compared with the C group, the thymus index was notably decreased, while the spleen index was increased (*p* < 0.05). Conversely, the thymus index in the CL group showed a significant increase, and the spleen index exhibited a significant decrease compared with the CP group after treatment with LNT (Figure 3B) (*p* < 0.05). Furthermore, no significant difference was observed between the C and L groups (*p* > 0.05). These findings suggest that LNT prevented weight loss and restored injury to the thymus and spleen in mice induced by CP.

### 2.3. Effects of LNT on Serum Cytokine Contents in LNT-Treated Mice

**Figure 4:**
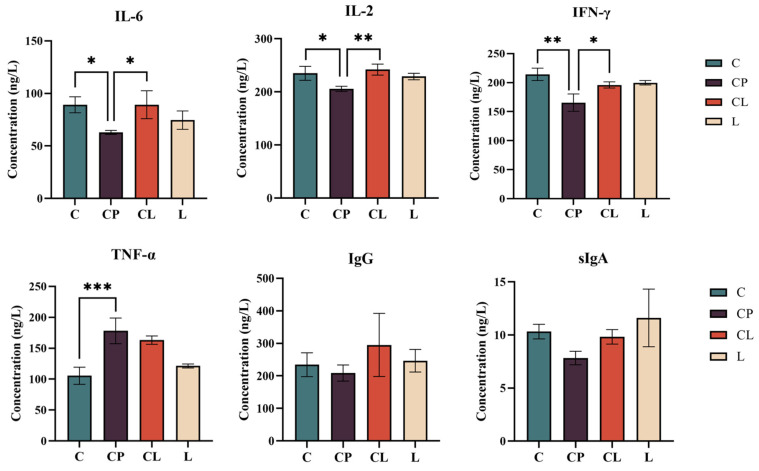
Cytokines in serum and *sIgA* in the ileum: The concentration of IL-6, IL-2, IFN-γ, TNF-α, and IgG in serum and sIgA in intestinal tract. Data are presented as the mean ± SD and analyzed by one-way ANOVA with Tukey’s multiple comparisons. * *p* < 0.05, ** *p* < 0.01, *** *p* < 0.001.

The levels of IL-2, IL-6, TNF-α, IFN-γ, and IgG in serum were assessed using ELISA. Additionally, sIgA levels in the ileum were also measured using the same method (Figure 4). However, in the CP group, a contrasting effect was observed: serum levels of IL-2, IL-6, and IFN-γ were found to be reduced compared with those in the C group. (*p* < 0.05). Besides, the level of IgG in serum and sIgA in the ileum had a downward trend, but the difference was not significant. The CL group significantly increased serum IL-6, IL-2, and IFN-γ levels as compared with the CP group (*p* < 0.05). The level of IgG in serum and sIgA in the ileum had an upward trend, though not statistically significant. The level of TNF-α in serum was increased remarkably in the CP group compared with the C group, and the CL group could alleviate the phenomenon could alleviate the above-mentioned phenomenon (*p* > 0.05).

### 2.4. The Results of Histopathology

**Figure 5:**
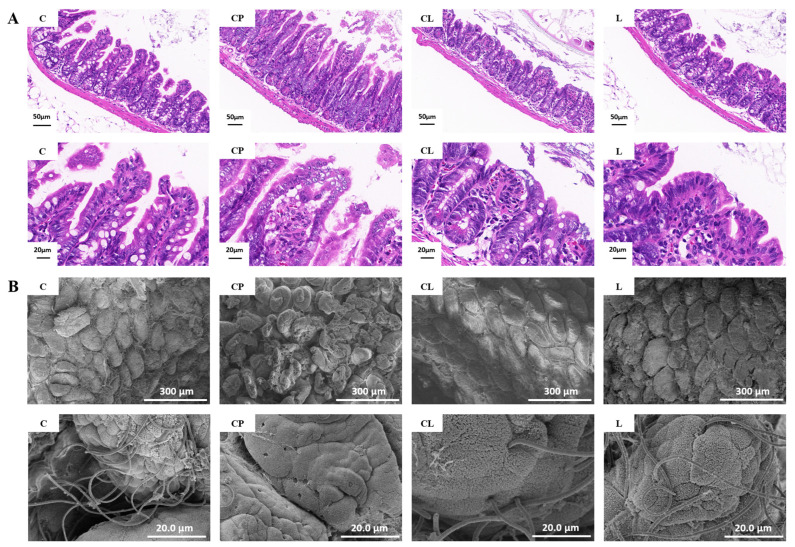
Pathological examination of ileum: (**A**) Representative histological images of ileum tissues by H&E staining. Scale bar: 50 μm/20 μm. The intestinal villi of the C group were normal and neatly arranged. The CP group exhibited significant damage to the intestinal wall, destruction of the structure of intestinal villi, and a looser arrangement. The clarity and structure of the intestinal villi in the CL group improved, with a closer arrangement of the villi. (**B**) SEM images of ileum tissues. Scale bar: 300 μm/20 μm.

The intestinal mucosa was examined using a light microscope on the tissue of the terminal ileum (Figure 5A). As is shown in Figure 5A, the intestinal villi in the C group appeared normal and were well organized, with the structure of the intestinal mucosa and epithelial cells intact in the small intestine. In comparison with the C group, after the administration of CP, the CP group exhibited significant damage to the intestinal wall, destruction of the structure of intestinal villi, and a looser arrangement. Following the intervention of LNT, the morphology of intestinal villi in the CL group gradually reverted to a palisade-like structure. The clarity and structure of the intestinal villi improved, with a closer arrangement of the villi. There was no significant difference in ileum morphology between the L group and the C group. Our results suggest that LNT could alleviate the intestinal damage that was caused by CP and protect the integrity of the intestinal structure.

As is shown in Figure 5B, the ileum was further assessed using a scanning electron microscope (SEM). At a scale of 300 μm, the integrity of intestinal villi in the CP group was compromised, exhibiting signs of damage to their surfaces. Compared with the CP group, the CL group has better cell completeness and narrower intercellular spaces. The intestinal microvilli were sparser and smoother in the CL group than in the CP group, meaning that treatment with LNT reduced this disruption. In comparison with the C group, the intestinal villi surface structure of the L group exhibited no significant changes, and the integrity of the intestinal villi remained intact. This demonstrates that LNT alone did not induce mechanical damage to the intestinal tract of normal mice. At a scale of 20.0 μm, it was observed that the CP group had reduced colonization of *Segmented Filamentous Bacteria* (SFB) due to immunosuppression, leaving more pores on the intestinal mucosal surface. The CL group significantly restored the colonization of this bacterium (Figure 5B).

### 2.5. Effect of LNT on Gene Expression in CP-Treated Mice

**Figure 6:**
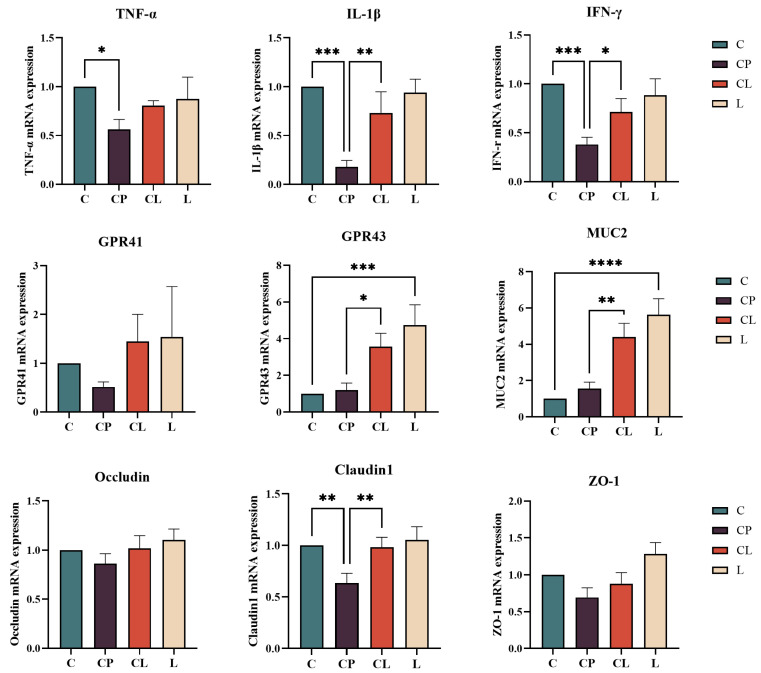
Rt-qPCR results of ileum: The mRNA expression of inflammatory cytokines in ileum, including TNF-α, IL-1β, and IFN-γ. The mRNA expression of GPR43, GPR41, and MUC2. The mRNA expression of intestinal tight junction proteins Occludin, Claudin1, and ZO-1. Data are presented as the mean ± SD and analyzed by one-way ANOVA with Tukey’s multiple comparisons. * *p* < 0.05, ** *p* < 0.01, *** *p* < 0.001, **** *p* < 0.0001.

In Figure 6, RT-qPCR analysis displayed that CP-induced mice had a lower expression of related genes, such as TNF-α, IL-1β, IFN-γ, and Claudin 1, than that in the normal group (*p* < 0.05). The CL group increased the related gene expression in comparison with the CP group (*p* < 0.05). Besides, the expression of GPR41, Occludin, and ZO-1 increased in the CP group and reversed in the CL group compared with the CP group (*p* > 0.05). Surprisingly, no matter which group, they changed the expression of GPR43 and MUC2 to a higher level. In the L group, intestinal mRNA expression levels of GPR41, GPR43, and MUC2 were also increased after administration of LNT alone. In general, LNT could promote the related genes of cytokines (TNF-α, IL-1β, and IFN-γ) to change intestinal function. Meanwhile, both CP and LNT could change the expression of GPR43 and MUC2.

### 2.6. LNT Modulates the Structure of the Gut Microbiota in CP-Treated Mice

**Figure 7:**
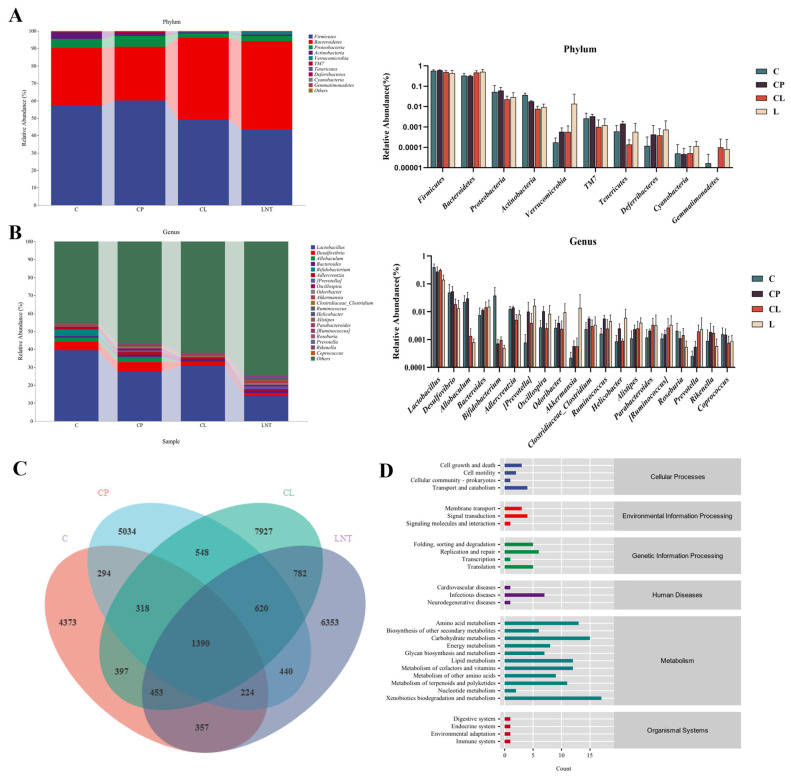
Gut microbiota structure changes based on 16S rRNA gene sequencing: (**A**,**B**) Species diversity composition of intestinal flora at phylum and genus levels. (**C**) Venn diagram of species differences between the four groups. (**D**) Enrichment analysis of KEGG metabolic pathways related to changes in gut microbiota.

Bioinformatics analyses of the microbiome were conducted using QIIME2 version 2019.4 [26], with minor adjustments made in accordance with the official tutorials (https://docs.qiime2.org/2019.4/tutorials/, accessed on 15 August 2022). In this experiment, CP treatment significantly changed the gut microbiota of mice. As shown in Figure 7A, we enumerate the changes in species composition at the top 10 phylum levels. The results indicate that the abundance of *Bacteroidetes* has decreased, whereas the abundances of *Firmicutes* and *Proteobacteria* have increased following treatment with CP. Conversely, under LNT treatment, *Bacteroidetes* increased, while both *Firmicutes* and *Proteobacteria* decreased compared with the CP group (Figure 7A). In Figure 7B, we present the species composition of flora at the top 20 genus levels. Following CP treatment, *Lactobacillus*, *Bifidobacterium*, and *Roseburia* exhibited a downward trend, which was restored by LNT treatment. Other bacterial groups demonstrated an upward trend after CP administration, while the composition of intestinal flora was partially restored following LNT treatment (Figure 7B). In comparison with group C, the relative abundance of both phylum and genus in group LNT exhibited an upward trend, indicating that LNT may enhance the abundance of gut microbiota.

**Figure 8:**
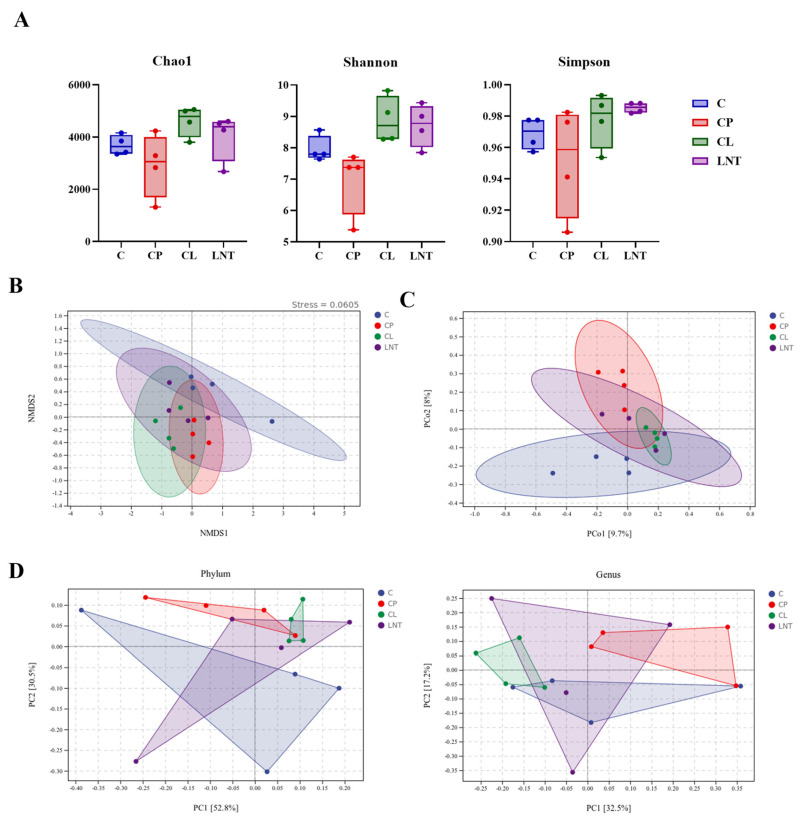
Diversity index results based on 16S rRNA gene sequencing: (**A**) The Alpha diversity index analysis of gut microbiota included Chao1, Shannon, and Simpson. (**B**) Nonmetric multidimensional scaling (NMDS) analysis. (**C**) Principal coordinate analysis (PCoA) of the microbiota based on Bray–Curtis distance. (**D**) Orthogonal partial least squares discrimination analysis (OPLS-DA) at both the phylum and genus levels.

In the CP group, the Chao1, Shannon, and Simpson indices were lower than those in the C group. The CL group adjusted the microflora structure, restoring the typical α-diversity indices to levels comparable to those of the C group (Figure 8A). The alterations in gut microbiota structure were further validated by the distinct clustering of the four groups observed in the principal coordinates analysis (PCoA) scatter diagram and the nonmetric multidimensional scaling (NMDS) analysis (Figure 8B,C). Additionally, orthogonal partial least squares discrimination analysis (OPLS-DA) at both the phylum and genus levels revealed distinct clusters within the gut microbiota (Figure 8D). Following the administration of CP, a shift in the microbial community structure was observed, which was partially reversed by LNT treatment. Notably, the LNT group also induced changes in the colony structure of the C group.

## 3. Discussions

Cyclophosphamide, a frequently used chemotherapeutic agent in clinical settings, is commonly employed in the treatment of tumors and autoimmune conditions. Despite its therapeutic benefits, the drug is associated with notable side effects, most notably immunosuppression and intestinal dysfunction. Previous research has demonstrated that lentinan polysaccharide possesses antioxidant, anti-radiation, and immune-enhancing properties [18,27]. This study investigated the immunomodulatory effects of LNT on CP-induced immunosuppression, with a particular focus on the impact of LNT on CP-induced changes in intestinal flora and function.

LNT was obtained from *Lentinus edodes* by water extraction and alcohol precipitation. The carbohydrate content of LNT was measured at 73.43%, with glucose, galactose, and D-mannose identified as the top three monosaccharides in descending order. Additionally, the protein content was found to be 17.17%, and the galacturonic acid content was recorded at 3.03%. Consequently, the next step is to investigate the bioactive role of carbohydrates as the primary components of LNT. The spleen and thymus play crucial roles as immune organs, and the indices of these immune organs reflect the functioning of innate immunity to some degree [28]. In our study, we found that CP can reduce the thymus index in mice, which is consistent with previous studies [29]. Furthermore, the administration of cyclophosphamide results in a higher spleen index, while the LNT treatment reversed the indices induced by CP. Our research results are consistent with those in previous research reports [30].

Cytokines are crucial in both mediating and regulating the immune response. IL-6, a multifunctional cytokine, significantly influences the regulation of immune activity [31]. IFN-γ stimulates the production of T cells, activates macrophages, and regulates the balance between Th1 and Th2 cells [32]. On the other hand, IL-2 is a multifunctional cytokine that supports T cell proliferation and triggers the development of Treg cells [33]. TNF-α is a potent pro-inflammatory cytokine that plays a crucial role in the immune response to inflammation. Our study revealed that treatment with LNT resulted in increased levels of IL-6, IFN-γ, and IL-2 in the serum supernatants of mice when compared with CP. Additionally, there was a noticeable upward trend in serum IgG levels following LNT treatment. Secretory IgA (sIgA) is produced by intestinal plasma cells and plays a crucial role in maintaining the integrity of the intestinal barrier [34]. It serves as an indicator of the functionality of the intestinal immune barrier to a certain extent [35]. Selective colonization of E. coli in the gut of germ-free animals has been shown to produce sIgA [36]. In instances of immunosuppression in our research, both the composition and the quantity of gut microbiota are altered, leading to a reduction in sIgA levels. Following treatment with LNT, these alterations were reversed, and similar functionality was observed in the unmolded group. These findings suggested that LNT has the potential to effectively alleviate the immunosuppression caused by CP.

The integrity of the intestinal mucosa plays an important role in the maintenance of intestinal mucosal barrier function, which prevents gut microbes and their toxins from migrating to extra-intestinal tissues and organs [37]. The analysis of our HE results revealed that mechanical injury occurred in the intestine following CP modeling, with LNT demonstrating a significant improvement in reducing this injury. Additionally, SEM results indicated a notable decrease in *SFB* colonization in the ileum of mice, accompanied by the presence of significant cavities on the surface of the villi, leading to mechanical damage in the ileum. However, administration of LNT successfully restored *SFB* colonization and mitigated the mechanical damage. Notably, SFB has been identified as a potent inducer of T helper 17 (Th17) cells, which play a crucial role in host defense against microbial pathogens [38].

Rt-qPCR results revealed that CP significantly decreased the expression levels of TNF-α, IL-1β, IFN-γ, and Claudin1 in the ileum when compared with the C group. Following LNT intervention, there was a notable increase in the expression of TNF-a, IL-1β, IFN-r, and Claudin1. In comparison with group C, CP was found to lower the expression of GPR41, Occludin, and ZO-1 in the ileum. LNT could restore these abnormal indexes, though the difference was not statistically significant. TNF-α, IL-1β, and IFN-γ are critical cytokines that play significant roles in the immune response. During pathogen invasion, TNF-α and IL-1β facilitate the recruitment and activation of inflammatory cells, while IFN-γ enhances cellular immune responses by promoting the activation of macrophages and their antigen presentation capabilities. In the context of CP-induced immunosuppression, the cytotoxic effects of CP lead to a reduction in the secretion of these cytokines, which significantly increases the body’s susceptibility to pathogens. The application of LNT makes up for the above cytokine defects in the application of CP. Occludin and Claudin-1, as tight junction proteins on the intestinal surface, directly affect the permeability of the intestinal tract and play an important role in maintaining the normal barrier function of the intestinal tract [39]. We propose that Occludin, Claudin-1, and sIgA play significant roles in enhancing the recognition of and immunity to pathogen invasion, with Occludin and Claudin-1 contributing to the mechanical barrier, while sIgA is associated with the mucosal immune barrier. In our study, MUC2 always showed an upward trend. MUC2, a significant gel-forming mucin, is secreted by intestinal epithelial goblet cells and plays a crucial role in transmitting immunomodulatory signals to reduce the immunogenicity of intestinal microorganisms. Interestingly, certain strains, like *Shigella* and *Sobacterium nucleatum*, have been found to increase the expression of MUC2 [40,41,42]. This means there is a two-way regulation between the MUC2 and the gut bacteria. In our 16S sequence detection results, the abundance of *Shigella* bacteria in the CP, CL, and LNT groups was significantly higher than that in the C group, resulting in a notable increase in MUC2 mRNA expression. Changes in the quantity and proportion of flora stimulated alterations in the short-chain fatty acid (SCFAs) content in the intestine, leading to increased mRNA expressions of the SCFAs receptors GPR41 and GPR43. Simultaneously, the small intestine’s epithelial cells respond to short-chain fatty acids (SCFAs) through the activation of the extracellular signal-regulated kinase 1/2 and p38 mitogen-activated protein kinase signaling pathways by GPR41 and GPR43. This activation subsequently leads to the production of chemokines and cytokines, which play a crucial role in immune responses [43].

This research employed 16S rRNA gene sequencing to assess the diversity and composition of microbiota. To evaluate the complexity of species diversity in gut microbiota, α-diversity was measured using various indices, such as Chao1 (https://www.genescloud.cn/home, accessed on 20 October 2022), Shannon (https://www.genescloud.cn/home, accessed on 20 October 2022), and Simpson (https://www.genescloud.cn/home, accessed on 20 October 2022). The findings indicated that CP led to a reduction in α-diversity, implying a decline in both the abundance and diversity of the gut microbial community, aligning with the observations made by Chen et al. [44]. KEGG level 2 analysis of the changes in the gut microbiota showed that the alterations were associated with various anabolic pathways.

As is widely recognized, numerous microbial communities within the intestines function as a biological barrier for the gut [45]. Intestinal CD4^+^ T lymphocytes can be differentiated into functional subtypes with regulatory or effector functions through the *Bacteroidetes*, thus playing a role in protecting the gut [46]. The *Proteobacteria* phylum contains various pathogenic microbes like *Helicobacter* and *Salmonella*. An increase in *Proteobacteria* levels can indicate a disruption in the gut microbiota and serve as a potential diagnostic factor for diseases [47]. We found that, at the phylum level, following LNT treatment, there was a decrease in the abundance of *Firmicutes* and *Proteobacteria*, while *Bacteroidetes* increased. This result indicates that LNT may enhance the dysbiosis of microbiota in mice with suppressed immunity and elevate the concentration of probiotics. At the genus level, the composition of *Lactobacillus*, *Bifidobacterium*, and *Roseburia* in the intestinal tract of the CP group decreased following the administration of CP. In contrast, the bacterial composition in the CL group showed recovery after the administration of LNT. It is well established that *Lactobacillus* and *Bifidobacterium*, which are among the most commonly utilized probiotic agents [48], play significant roles in mitigating intestinal inflammation and enhancing intestinal immunity and barrier function [49]. Recent studies have indicated that *Roseburia* may protect against colorectal tumorigenesis through the production of butyrate [50]. Additionally, the treatment of LNT further enhances the abundance of bacteria associated with cancer treatment; for instance, the increased presence of *Alistipes* is likely to have a beneficial effect on cancer therapy [51].

In conjunction with these findings, we demonstrate that LNT can not only mitigate CP-induced immunosuppression and intestinal dysfunction but also regulate the imbalance of intestinal microbiota induced by CP while enhancing the abundance of bacteria associated with adjuvant cancer therapy and treatment.

## 4. Materials and Methods

### 4.1. Materials and Reagents

The fresh *Lentinus edodes* were collected from Harbin (Harbin, Heilongjiang, China). CP was purchased from Suri Pharmaceutical Chemical Co., LTD (Suzhou, Jiangsu, China). ELISA kits of interferon-γ (*IFN-γ*), interleukin-2 (*IL-2*), interleukin-6 (*IL-6*), tumor necrosis factor-α (*TNF-α*), immunoglobulin G and sIgA (*IgG* and *sIgA*) were purchased from Andy Huatai Technology Co., Ltd. (Beijing, China). TRIzol^®^ Reagent (9108Q), PrimeScript RT reagent Kit (RR047A), and TB Green^®^ Premix Ex Taq™ (RR820A) were purchased from Takara Baori Doctor Material Technology Co., Ltd. (Beijing, China).

### 4.2. Polysaccharide Preparation and Characterization

#### 4.2.1. Extraction of LNT

Based on the previous literature on the extraction methods of fungal plants [52], we adjusted the partial extraction methods. The specific methods are described below. The extraction process of lentinan is shown in Figure 1A. The *Lentinus edodes* were crushed into powder after being dried. The residue was extracted twice with 90 °C water for 2 h each time. After filtration and concentration by rotary evaporator, the filtrate was precipitated with 75% ethanol (final concentration) overnight. The precipitate was collected after centrifugation at 4000× *g* for 15 min and redissolved using sterilized distilled water. Deproteinization was performed with Sevage reagent and decolorized with macroporous resin. The crude product was obtained after vacuum freeze-drying, named LNT.

#### 4.2.2. Determination of Total Carbohydrate, Protein, and Galacturonic Acid Contents

The total carbohydrate contents were analyzed by the Phenolsulfuric acid method using dextrose as a standard [53]. Specific methods are as follows. Accurately weigh the dried D-glucose in a test tube and dilute it with deionized water to achieve concentrations of 0.01, 0.02, 0.04, 0.06, and 0.08 mg/mL. For each test tube solution, take 1 mL and add 1.0 mL of 5% phenol solution and 5.0 mL of concentrated sulfuric acid. After vortexing, allow the reaction to proceed at room temperature for half an hour. The absorbance at 490 nm was measured using the solutions without D-glucose as the control. Each sample is repeated three times.

M-hydroxybiphenyl colorimetric method was utilized to quantify the Galacturonic acid (GalA) content in LNT [54]. M-hydroxybiphenyl colorimetric method was utilized to quantify GalA in LNT. Weigh 0.15 g m-hydroxybiphenyl, dissolve it in 5 mg/mL sodium hydroxide solution, and adjust the concentration to 1.5 mg/mL. Then, add 20 μL of LNT solutions into a 1 mL centrifuge tube, add 60 μL of sodium tetraborate/sulfuric acid solution, mix it with a vortex mixer, heat it in a boiling water bath for 5 min, and put it in an ice water bath. Then, 100 μL of the 1.5 mg/mL m-hydroxybiphenyl solution was added to the mixture and shaken for an additional 5 min. A total of 200 μL of the mixture was added to a 96-well plate, and the absorbance was measured at 520 nm. Prepare a standard GalA solution of 0–60 μmol/mL in the same way and measure the absorbance at 520 nm according to the above method. Each sample is repeated three times.

Protein content was detected using the BCA protein assay kit (BL521A, Biosharp) according to the manufacturer’s instructions. Briefly, liquid A and liquid B in the reagent are mixed at a ratio of 50:1. The standard solution is then diluted to a concentration ranging from 0.025 mg/mL to 2 mg/mL. In a 96-well plate, 20 μL of each of the standard diluents and sample solutions was added to each well, followed by the addition of 200 μL of the working solution. After incubating at 37 °C for 30 min, the absorbance was measured at 562 nm. Each sample is repeated three times.

#### 4.2.3. Determination of Monosaccharides Composition

LNT (5 mg) was hydrolyzed with 2 M trifluoroacetic acid (TFA, 3 mL) at 120 °C for 4 h. Next, the hydrolysate was derivatized with 3-methyl-1phenyl-2-pyrazolin-5-one (PMP) and then analyzed by a previously reported HPLC method, with some modification [55,56]. The samples or monosaccharide standards were mixed with an equal volume of 0.6 M NaOH aqueous solution (250 μL) and 0.4 M PMP methanol solution. The derivatization proceeded at 70 °C for 1 h after vortex blending. Then, the reaction mixture was neutralized with 500 μL of an HCl solution (0.3 M), and the excess PMP was removed by chloroform three times. Then, the filtrate of the aqueous layer was evaluated by a Daojin LC-20AD HPLC instrument with an Xtimate C18 column (4.6 × 200 mm 5 µm). A potassium dihydrogen phosphate solution (50 mM) and acetonitrile in a ratio of 83:17 (*v*/*v*) were used as eluent, and the flow rate was maintained at 1.0 mL/min.

#### 4.2.4. Morphological Observations

The LNT was affixed to the sample stage using double-sided conductive tape, and images were captured at various magnifications with a Phenom Pro scanning electron microscope (SEM, HITACHI, Tokyo, Japan). Atomic force microscopy (AFM) shooting was improved based on previous research methods [57]. The LNT was fully dissolved in pure water at a concentration of 30 µg/mL, and 30 µL of this solution was deposited onto a freshly cleaved mica surface to obtain atomic force microscopy (AFM) images. Following air drying, the morphology was examined using a Dimension Edge AFM (Bruker, Penang, Malaysia).

### 4.3. Animal Experiments

#### 4.3.1. Animals and Experimental Design

Twenty-four male Kunming mice (inbred, 6–8 weeks old), weighing 18~22 g, were provided by the Liaoning Changsheng Biotechnology Co., Ltd. (Benxi, Liaoning, China, license number SCXK (Liao)2020-0001). After fasting for 12h, the mice were anesthetized with isoflurane in an anesthesia box and then euthanized with eye blood collection. Throughout the whole experiment, the mice were reared at room temperature (25 ± 2 °C), humidity (55–60%), and a 12 h light–dark cycle with arbitrary water and food. The present study was conducted under the approval of the Institutional Animal Care and Use Committee of Northeast Agricultural University (Heilongjiang province, China) (SYXK (Hei) 2012–2067) in accordance with the Laboratory Animal Guideline for ethical review of animal welfare.

**Figure 9:**
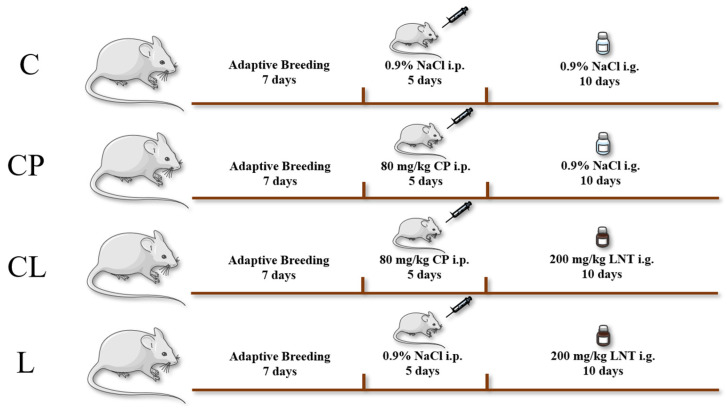
Animal and experimental design. Animals (n = 24) were divided into four experimental groups. From the 1st day to the 5th day, the intraperitoneal injection of mice was as below: Model group (CP) and LNT treatment group (CL): intraperitoneal injection of 80 mg/kg CP; NC group (C) and LNT administration alone group (L): intraperitoneal injection of 0.9% NS. From the 6th day to the 15th day, there were two LNT groups (CL and L): intragastric administration of 200 mg/kg LNT, and the other groups were as follows: intragastric administration of 0.9%NS.

Following a week of adaptive breeding, the mice were split into four groups at random, with each group containing 6 mice. From the 1st day to the 5th day, the intraperitoneal injection of mice was as below: Model group (CP) and LNT treatment group (CL): intraperitoneal injection of 80 mg/kg CP; NC group (C) and LNT administration alone group (L): intraperitoneal injection of 0.9% NS. From the 6th day to the 15th day, there were two LNT groups (CL and L): intragastric administration of 200 mg/kg LNT [19], and the other groups were as follows: intragastric administration of 0.9%NS (Figure 9). The mice were euthanized on the 17th day, and serum, spleen, thymus, and ileum were collected for subsequent experiments.

#### 4.3.2. Immune Organs Indices

After administration, the mice were anesthetized with isoflurane and euthanized by eye blood collection. The thymus gland and spleen were removed and weighed. Immune organ index was determined using the following formula: Thymus or spleen index = weight of thymus or spleen/body weight × 1000.

#### 4.3.3. Measurements of Cytokines in Serum and sIgA in the Ileum

Serum was obtained by centrifugation after the blood was left for 2 h at 4 °C. The ileum samples were crushed and centrifuged at 4 °C 3000 rpm for 5 min to obtain the supernatant. The concentrations of cytokines in the serum and sIgA in the ileum were determined by the ELISA kit according to the instructions of the manufacturer.

#### 4.3.4. Real Time-qPCR (RT-qRCR) Assay

The total RNA was extracted from the ileum using the Trizol reagent. The RNA was performed at 37 °C for 15 min and 85 °C for 5 sec to prepare cDNA, which was then stored at −80 °C for use. Real-time PCR was carried out using a LightCycle 96 real-time PCR system and a real-time PCR master mix (SYBR Green) reagent kit (Takara, China). Custom-made primers are present in Table 2. The conditions for real-time PCR were 95 °C for 5 min and then 45 cycles at 95 °C for 10 s, 60 °C for 30 s, and 95 °C for 15 s, 60 °C for 1 min, 95 °C for 15 s. Relative mRNA expression was calculated by the 2^−△△^CT method.

#### 4.3.5. Histopathology

The ileum was collected after washing and fixed in a 10% formalin solution. They were then sectioned and stained with H&E.

#### 4.3.6. Scanning Electron Microscopy (SEM)

Scanning electron microscopy (SEM) was performed as previously described. Briefly, samples were fixed with 2.5% glutaraldehyde and rinsed in 0.1 M sodium phosphate buffer. After dehydration and replacement with ethanol and tert-butyl alcohol, the mixture was dried at −20 °C for 30 min, and then dried in ES-2030 (HITACHI) for 4 h. Samples were metal coated and examined with SU8010 field emission scanning electron microscope (HITACHI, Tokyo, Japan).

#### 4.3.7. 16S rRNA Gene Sequencing of the Gut Microbiota in Fecal Samples

The total DNA was extracted from the fecal samples following a previously reported method, with certain modifications [58]. Briefly, the OMEGA Soil DNA Kit (M5636-02) (Omega Bio-Tek, Norcross, GA, USA) was used following the manufacturer’s recommendations. The bacterial 16S rRNA gene V3-V4 region was amplified by PCR using the forward primer 338F (5′-ACTCCTACGGGAGGCAGCA-3′) and the reverse primer 806R (5′-GGACTACHVGGGTWTCTAAT-3′). PCR amplicons were purified with Vazyme VAHTSTM DNA Clean Beads (Vazyme, Nanjing, China) and quantified using the Quant-iT PicoGreen dsDNA Assay Kit (Invitrogen, Carlsbad, CA, USA). Sequence data analyses were mainly performed using QIIME2 version 2019.4 and R packages (v3.2.0).

### 4.4. Statistical Analysis

All results were confirmed in at least three independent experiments. Results were expressed as mean ± SD. All data were analyzed by one-way ANOVA with Tukey’s post-test. Significance was defined as *p* < 0.05.

## 5. Conclusions

In summary, the LNT we extracted is a brown-yellow, hygroscopic powder with a total carbohydrate content of 73.43% (*w*/*w*), a protein content of 17.17% (*w*/*w*), and a galacturonic acid content of 3.03%. In vivo experiments confirmed that LNT can regulate cyclophosphamide (CP)-induced immunosuppression, restore cytokine levels in serum, and reinforce the intestinal mucosal barrier. Additionally, LNT has been shown to normalize the gut microbiota structure affected by CP, particularly by enhancing the growth of bacteria associated with cancer treatment. These findings establish a foundation for the potential development of LNT as functional foods and drugs with immunomodulatory properties. However, the precise mechanism by which LNT exerts its immunomodulatory effects through gut microbiota remains unclear and warrants further investigation.

## Figures and Tables

**Table 1:** Identification of monosaccharides by HPLC.

Number	Retention Time	Compound	Area	Concentration μg/mL	Percentage %
1	15.768	D-Mannose	326,336	11.219	4.728
2	20.149	Ribose	125,820	3.898	1.823
3	20.918	L-Rhamnose	4197	0.199	0.061
4	25.249	D-Glucuronic acid	122,023	4.705	1.768
5	33.552	Glucose	5,252,654	202.539	76.104
6	38.492	Galactose	805,757	24.538	11.674
7	41.975	L-Arabinose	60,595	1.539	0.878
8	47.026	Fucose	204,572	6.873	2.964

**Table 2:** Primers used for Rt-PCR analysis of genes.

Genes	Forward (5′-3′)	Reverse (5′-3′)
IL-1β	CACTACAGGCTCCGAGATGAACAAC	TGTCGTTGCTTGGTTCTCCTTGTAC
IFN-γ	CTTGAAAGACAATCAGGCCATC	CTTGGCAATACTCATGAATGCA
TNF-α	ATGTCTCAGCCTCTTCTCATTC	GCTTGTCACTCGAATTTTGAGA
GPR41	CCACACTGCTCATCTTCTTCGTCTG	ACGGACTCTCACGGCTGACATAG
GPR43	CTGTATGGAGTGATCGCTGCTCTG	CTGCTCTTGGGTGAAGTTCTCGTAG
MUC2	CGAGCACATCACCTACCACATCATC	TCCAGAATCCAGCCAGCCAGTC
Claudin-1	AGATACAGTGCAAAGTCTTCGA	CAGGATGCCAATTACCATCAAG
Occludin	TGCTTCATCGCTTCCTTAGTAA	GGGTTCACTCCCATTATGTACA
ZO-1	CTGGTGAAGTCTCGGAAAAATG	CATCTCTTGCTGCCAAACTATC
GAPDH	CATCTTCCAGGAGCGAGACC	TCCACCACCCTGTTGCTGTA

## Data Availability

All relevant data are within the manuscript and its Supplementary Materials.

## Supplementary Materials

The following supporting information can be downloaded at https://www.mdpi.com/article/10.3390/ijms252212432/s1.

## Abbreviations

LNT: lentinan; CP, cyclophosphamide; GalA, galacturonic acid; IL-6, interleukin 6; IL-2, interleukin 2; IFN-γ, interferon-γ; TNF-α, tumor necrosis factor α; IgG, immunoglobulin G; sIgA, secretory immunoglobulin A; IL-1β, interleukin 1β; GPR41, G protein-coupled receptor 41; GPR43, G protein-coupled receptor 43; MUC2, Mucin2; ZO-1, zonula occludens-1; SEM, scanning electron microscopy; SFB, *Segmented Filamentous Bacteria*; NMDS, nonmetric multidimensional scaling; PCoA, principal coordinates analysis; OPLS-DA, orthogonal partial least squares discrimination analysis.
