# Supplementary material for: Extraction and Identification of Polysaccharide from Lentinus edodes and Its Effect on Immunosuppression and Intestinal Barrier Injury Induced by Cyclophosphamide
Source: Int J Mol Sci. 2024 Nov 19;25(22):12432. doi: 10.3390/ijms252212432 (PMC11594469; doi:10.3390/ijms252212432)
Supplement: Supplementary file 1 [file ijms-25-12432-s001.zip › Supplementary Materials File S1/4.Materials and Methods of HPLC.docx]

**Determination of monosaccharides composition**

LNT (5 mg) was hydrolysed with 2 M trifluoroacetic acid (TFA, 3 mL) at 120℃ for 4 h. Next, the hydrolysate was derivatized with 3-methyl-1phenyl-2-pyrazolin-5-one (PMP), and then analyzed by a previously reported HPLC method, with some modification. The samples or monosaccharide standards were mixed with an equal volume of 0.6 M NaOH aqueous solution (250 μL) and 0.4 M PMP methanol solution. The derivatization proceeded at 70 ◦C for 1 h after vortex blending. Then, the reaction mixture was neutralized with 500 μL of an HCl solution (0.3 M), and the excess PMP was removed by chloroform three times. Then, the filtrate of the aqueous layer was evaluated by a Daojin LC-20AD HPLC instrument with an Xtimate C18 column (4.6*200mm 5um). Potassium dihydrogen phosphate solution (50 mM) and acetonitrile in a ratio of 83:17 (v/v) were used as eluent and the flow rate was main­ tained at 1.0 mL/min
