# Supplementary figures and images for: Extraction and Identification of Polysaccharide from Lentinus edodes and Its Effect on Immunosuppression and Intestinal Barrier Injury Induced by Cyclophosphamide
Source: Int J Mol Sci. 2024 Nov 19;25(22):12432. doi: 10.3390/ijms252212432 (PMC11594469; doi:10.3390/ijms252212432)

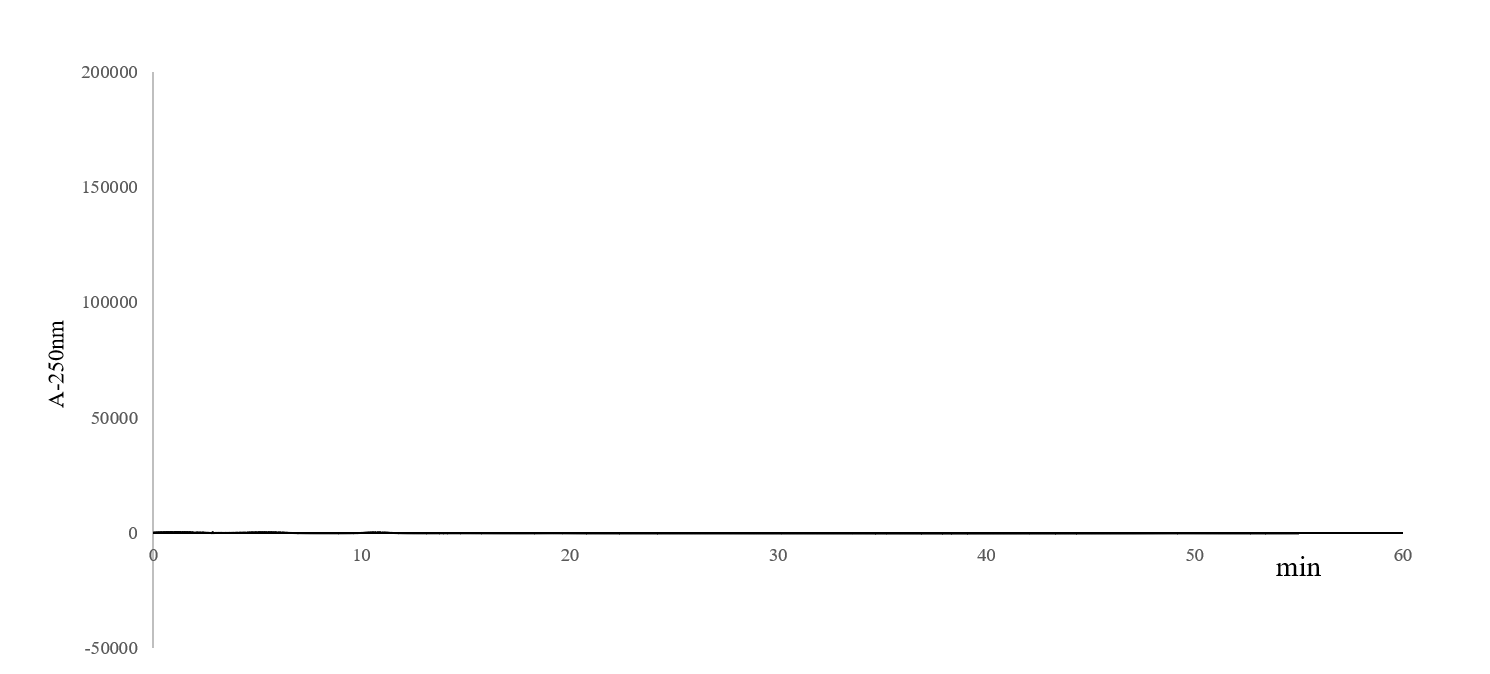

Supplement: Supplementary file 1 [file ijms-25-12432-s001.zip › Supplementary Materials File S1/1.Blank solution.tif]

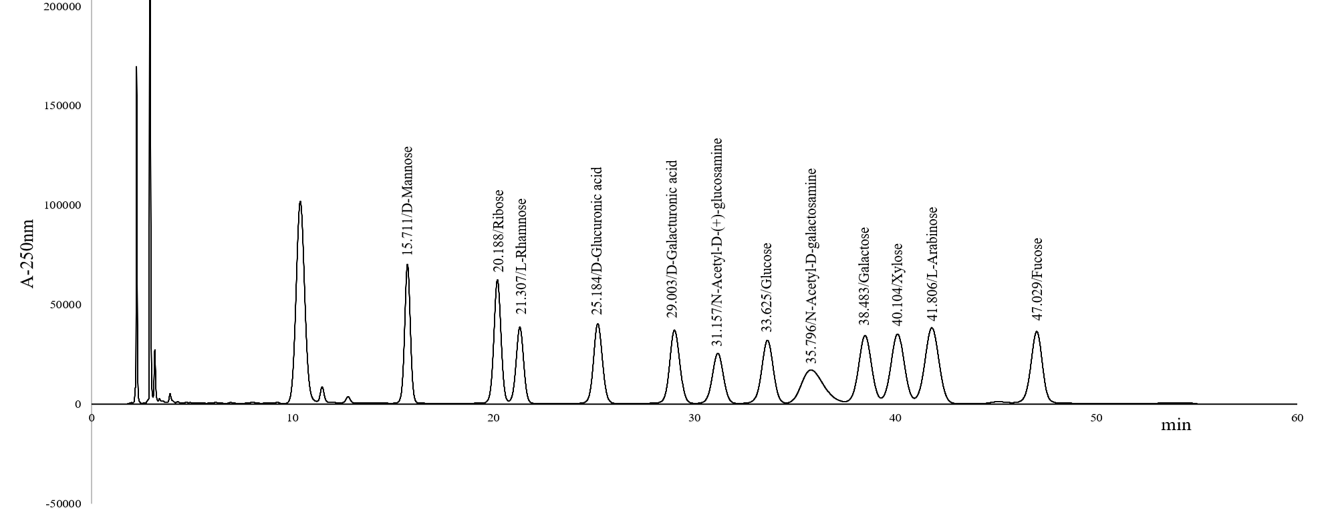

Supplement: Supplementary file 1 [file ijms-25-12432-s001.zip › Supplementary Materials File S1/2.Contrast solution.tif]

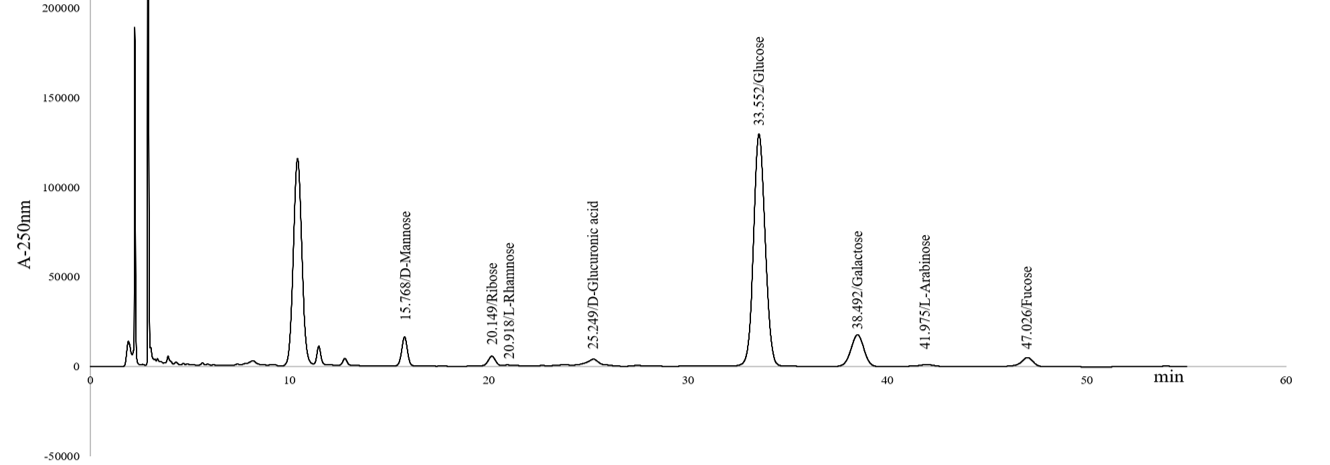

Supplement: Supplementary file 1 [file ijms-25-12432-s001.zip › Supplementary Materials File S1/3.Sample solution.tif]
